# The Care of the Navel

**Published:** 1895-09

**Authors:** 


### Selections and Abstracts.

### THE CARE OF THE NAVEL.

DOKTOR (*Archiv fur Gynakologie*, Bd. XLV, H. 3) reports his experience relative to the treatment of the umbilicus in newborn infants, and the prevention of infections. In newborn infants the navel forms a columnar projection of the skin, on the top of which the cord is attached, a sharp line of demarcation, the navel ring, separating the cord from the skin. On its margin are numerous vessels that go to the border of Wharton's jelly, but do not enter into it. When the cord is ligated, its tissues lose their viability and must separate and fall away, leaving the wound covered with a living structure. We must regard the navel as a physiological wound of the abdomen of the newborn, its healing differing in no way from that of any other wound, the only peculiarity being the topography of the wound. In typical cases it heals by first intention. This small wound is especially liable to infection and resultant maladies, severe or light:

(*a*) Because of its condition, it is not merely a wound of the abdominal skin, but also of its wall, and in close proximity to the abdominal membrane (peritoneum), which is very susceptible to infection.

(*b*) The peculiarity that these great vessels lie free in this wound.

(*c*) The third great factor tending to infection is the disproportionately large mass of dead tissue,—the remains of the cord.

(*d*) This wound is peculiarly inclined to an excessive formation of granulations.

(*e*) The frequency of development of anomalies and aberrations of the umbilicus also predisposes it to disease.

According to Eros, 68 per cent of umbilical wounds do not heal in a normal manner; and of these cases 45 per cent suffer from fever. How often these cases terminate fatally is not known. In treating the navel, the aim is to obtain healing without infection. The ordinary method is to ligate the cord some eight or ten centimeters from the body and wrap it in an oiled rag after careful disinfection with sublimate solution, 1-1000, and then binding it to the abdomen with a bandage. At each bathing of the child the cord is washed, and if there be not much secretion, a new bandage is applied or a cotton pad is placed over the navel first. Too often cleanliness in the latter matter is neglected by the nurse. The author omitted the oiling of the rag in the above method as it hindered mummification. The temperature was taken twice daily. Iodoform was applied to the wound or, if needed, a weak carbolized wash. With the above treatment 35 per cent of cases had fever, and of these 16 per cent showed infection. The plan was then changed. The cord was removed as early as possible and all wetting omitted. The bandage was changed daily. After this 25.8 per cent showed a rise of temperature, and of these 10 per cent had infection. Further improvement resulted when efforts were made to hasten mummification of the cord. Ligatures applied closely to the belly were next tried, the stump being one centimeter long. This gave 11.88 per cent of fever, and 3.46 per cent of infection. As a general rule, fever occurring during the healing of the navel is due to infection, notwithstanding the failure of local symptoms, and especially the coincident frequent digestive disturbances cause no fever. The author summarizes the treatment of the umbilicus as follows:

(1) Cut the cord as close as possible.

(2) The bandage once applied, should not be changed except for good cause, and preferably the bath should be omitted.—*University Medical Magazine.*

